# How do medical and nursing students experience emotional challenges during clinical placements?

**DOI:** 10.5116/ijme.5a88.1f80

**Published:** 2018-03-27

**Authors:** Maria Weurlander, Annalena Lönn, Astrid Seeberger, Eva Broberger, Håkan Hult, Annika Wernerson

**Affiliations:** 1 Department of Learning, School of Education and Communication in Engineering Science (ECE), KTH Royal Institute of Technology, Stockholm, Sweden; 2 Department of Clinical Science, Intervention and Technology (CLINTEC), Division of Renal Medicine, Karolinska Institutet, Stockholm, Sweden; 3 Department of Neurobiology, Care Sciences and Society (NVS), Division of Nursing, Karolinska Institutet, Stockholm, Sweden

**Keywords:** Emotional aspects, student experiences, nursing education, medical education, narrative inquiry, qualitative analysis

## Abstract

**Objectives:**

To investigate which kinds of situations medical and nursing students found emotionally challenging during their undergraduate education, and how they managed their experiences.

**Methods:**

This study used an exploratory research design. We gathered qualitative data using an open-ended questionnaire distributed to students in the middle and at the end of their education. In total, 49 nursing and 65 medical students participated. Also, five students were interviewed individually to acquire richer data. Data were analysed using narrative thematic analysis.

**Results:**

Medical and nursing students experienced a range of situations during their undergraduate education that they found emotionally challenging, mainly during clinical placements. The students’ narratives concerned confronting patients’ illness and death, unprofessional behaviour among healthcare professionals, dilemmas regarding patient treatment, students relating to patients as individuals and not diagnoses, and using patients for their own learning. The narratives concerned both the formal and the hidden curriculum, i.e., what is included in the profession (confronting illness and death), and what is not (unprofessional behaviour among healthcare professionals). Students managed their experiences by talking to trusted peers or supervisors, and by getting used to these situations.

**Conclusions:**

Despite the different knowledge, experiences, and conditions for medical and nursing students, our findings suggest that their experiences of emotional challenges are similar. Support and opportunities to talk about these experiences are important. Teachers, supervisors, and students need to be aware that students might experience emotionally difficult situations, and that the students need time for reflection and support.

## Introduction

Healthcare education involves situations where students engage in interaction with other people. Learning to care for and help patients involves a variety of emotions. Students may experience both positive feelings, such as interest, joy and satisfaction, and negative feelings, such as sadness, guilt, anxiety, anger, and shame. Medical and healthcare students need to learn how to manage their own emotions as well as the emotions of their patients.^1^

Previous research has found that anatomy dissection and autopsies during the pre-clinical years evoked strong emotional reactions in many medical students and they reacted in different ways.^2^^-^^6^ Some students found the dissection or autopsy mostly interesting and important, although some of them felt uneasy. Other students had strong negative reactions and difficulties coping with the situation and subsequently found it hard to focus on learning.^2^^,^^6^ The clinical placements also provide students with learning situations that might cause strong emotional reactions. Witnessing the death of a patient can be a powerful emotional experience that students will probably remember for a long time.^7–10^ Furthermore, students have been found to experience a range of professionalism dilemmas during their clinical practice^11^^-^^14^, e.g., unprofessional behaviour from healthcare professionals, or abuse and discrimination directed towards students. Experiencing these kinds of professional dilemmas evokes emotions and may cause moral distress. The strong emotions that students may feel in the situations described above are likely to influence their learning.

Thus, students experience a range of emotions, both positive and negative, during their undergraduate education. The negative feelings are more likely to be troublesome and may hinder learning. The literature suggests that students have for the most part to manage emotionally challenging experiences by themselves.^2^^,^^10^^,^^23^ Different coping strategies may be developed in order to handle the situation; some students may distance themselves by trying to turn off their emotions; others may become overwhelmed with emotions and have difficulties in managing them. Students seem to struggle to find a balance between closeness and distance, i.e., in developing a professional attitude.^2^ When their clinical supervisors do not express emotions or explicitly notice students’ or patients’ emotional reactions, the message conveyed is that emotions should be ignored and emotional reactions are unwanted.^10^ Also, in addition to the lack of support from others, medical students may be reluctant to seek help and support when they are distressed, as they might be viewed as being weak.^24^

The literature reviewed above suggests that there are situations during undergraduate education that healthcare students might find emotionally challenging. However, we do not know enough about how students experience these situations, how they seek and receive support and what kind of support they receive, and what they learn from these experiences. It is, therefore, important to explore these issues further in order to inform researchers and healthcare educators. The present study aimed to investigate broadly which kinds of situations medical and nursing students found emotionally challenging during their undergraduate education. Our study complements previous research, since we take the student’s emotional experiences as a point of departure and enquire into any situation students found emotionally challenging. The research questions addressed in the study were: What kinds of emotionally challenging situation do medical and nursing students encounter during pre-clinical and clinical education? How did they manage their experiences?

### Conceptual framework

In our study, we considered emotion and cognition to be highly interrelated and in practice impossible to differentiate.^15^ Emotions influence our daily lives in many ways and help us to understand ourselves and others.^16^ Furthermore, memory formation is enhanced by emotions, and memories of emotional events are often well preserved over time.^17^ It is, therefore, important to deepen our understanding of the emotional challenges that students encounter during their education.

Healthcare students spend a large part of their education in clinical environments, and they are expected to learn theoretical knowledge, practical skills and professional attitudes during these placements. These dimensions of knowledge and competence represent the formal curriculum.^18^ Many situations in clinical work are emotionally challenging, and students need to manage their emotions in order to cope and subsequently to learn what is expected of the formal curriculum. If students do not learn how to manage their own and others’ emotions, in the long run, they might risk developing compassion fatigue or even burnout.^19^ Furthermore, being aware of one’s own and patients’ feelings is part of good patient care.^1^ Teachers, supervisors and other healthcare professionals are role models in this process and influence the meaning given to emotions in the particular practice.^16^^,^^20^ The way that clinical supervisors and other healthcare professionals acknowledge and deal with emotions becomes part of the “hidden curriculum”, i.e., the underlying assumptions of values, norms and attitudes of “how things are done” in a specific context, and how they influence everyday practice.^1^^,^^18^^,^^21^ The hidden curriculum within medical training has sometimes been found to contradict the formal curriculum, which is the competencies, knowledge, values, and norms explicitly expressed in the medical curriculum.^22^

## Methods

### Context of the study

The context of the study was a medical and nursing undergraduate curriculum at a Swedish university. In Sweden, both nursing and medical education are academic undergraduate programmes; nursing education lasts three years (180 ECTS credits), and medical education lasts 5.5 years (330 ECTS credits). On both programmes, students spend considerable time on clinical placements; in hospitals, primary healthcare settings and sometimes also in municipal care. On the medical programme, the clinical placements on hospital wards are often short, 1–2 weeks. By contrast, nursing students spend several weeks, usually five to six, on the same clinic or ward. Nursing students often have a dedicated clinical supervisor through their placement. Medical students, on the other hand, often have several supervisors during their short clinical placements. Thus, the conditions during the clinical placements for medical and nursing students are quite different. Furthermore, both programmes involved in this study have an integrated course module called “professional development” enabling students to learn how to act professionally in encounters with patients, their family members, and in cooperation with other health professionals. These modules include lectures, seminars and written tasks focusing on ethical, psychological and communication issues.

### Research design and participants

An exploratory research approach was chosen in order to investigate which kinds of situation students found emotionally challenging. As we wanted to acquire a broad overview, we chose to gather qualitative data using questionnaires with open-ended questions.^25^ As a complement to the questionnaires, we conducted individual semi-structured interviews. These interviews gave a more nuanced and in-depth understanding of students’ experiences of emotionally challenging situations.

We anticipated that all or most of the students would have experienced emotionally challenging learning situations by the end of their undergraduate studies. Therefore, the questionnaire was distributed to nursing and medical students in their final year, i.e., year three and six respectively. We also wanted to capture any experiences that students might have had during the first part of their education, so the questionnaire was also distributed to students during their second year (nursing students) and third year (medical students). In total, 49 nursing and 65 medical students chose to participate.

In addition, a selection of nursing and medical students was contacted by e-mail and asked to participate in individual interviews. In order to maximise variation, a purposive sample was used, and students were selected based on gender and ethnicity. About ten students, selected randomly from the course lists based on gender and ethnicity, were contacted. Five students – three medical and two nursing students – volunteered to be interviewed. One of the students was a man (medical student), and the others were women. One of the nursing students was of non-Swedish origin.

### Data collection

The questionnaire consisted of two open-ended questions that students answered anonymously using pen and paper. The students were asked to describe any situation they had experienced during their education that was emotionally challenging, what happened, how they felt, and how they dealt with the situation. The questions were deliberately broad since we wanted to capture any situation that students found emotionally challenging. They were also asked to describe whether they had received support from teachers or supervisors. The students answered the questions during ordinary lessons. We chose seminars which focused on reflection on professional aspects, i.e., during the education in professional development. The reason for this was that our investigation focused on emotionally challenging situations, which is something that students need to handle in order to develop as healthcare professionals.

The interviews were semi-structured and focused on students’ experiences of emotionally challenging learning situations, i.e., what they experienced, how they felt, how they dealt with the situation and what kind of support they received. During the interviews, the students were asked to share stories about emotionally challenging situations that they had encountered during their education. The interviews were conducted in Swedish, lasted between 18 and 51 minutes, and were conducted by the first author in a quiet room, either on the ward where the students were placed or in the department where the interviewer worked. The interviews were recorded and later transcribed verbatim. The quotes used in this paper were translated into English by the first author and later discussed and compared to the original wording by the research team.

The study followed ethical guidelines concerning research involving people.^26^ Participation in the study was voluntary, and the choice of taking part in the study did not in any way affect whether the students passed or failed their courses. Ethical approval was granted by the regional ethical board in Stockholm. The students involved were informed orally and in writing about the purpose of the study and their rights as participants. Consent was obtained in writing prior to data collection.

### Data analysis

Data from the interviews and open-ended questionnaires in the present study were narrative in nature, i.e., like short stories, and consequently, a narrative approach to data analysis was chosen.^27^^-^^29^ Humans often make sense of their experiences in the form of stories or narratives, and narrative methods are becoming more common in medical education.^28^^,^^30^ Polkinghorne^27^ describes two forms of narrative inquiries: analysis of narratives, and narrative analysis. In the former, narrative data is analysed analytically and results in themes or categories; in the latter, the data is analysed more holistically, and the result itself is in the form of narratives. In the present study, we have used the first form of narrative inquiry, i.e., our narrative data were analysed inductively using narrative thematic analysis.^29^ Not all answers to the questions in the questionnaire were narrative in character, and we chose to only include narrative data in the analysis since this data answered our research questions. Short answers, in a sentence or two, did not contribute enough to our understanding of students’ experiences of emotionally challenging situations, and were, therefore, excluded. Of the 114 student answers, 63 were narrative and were thus included in the analysis. Narrative methods are case-centered, and each narrative, consisting of a description of an event that students witnessed or participated in, was read as a whole. The interview transcripts were analysed in the same way: first, each transcript was read as a whole and then focusing on analysing the narratives described. All narratives, both from the questionnaire and interviews, were read several times before they were analysed regarding what happened, students’ emotional reactions, and how they dealt with the situation. Each narrative was then labelled, in an attempt to capture what the story communicated about the experience. Similar narratives were grouped, resulting in five themes. The themes were constructed across the individual narratives, thus representing different kinds of emotionally challenging experiences.

Trustworthiness of the study was established in several ways. Firstly, by investigator triangulation,^31^ where the members of the research team have different academic backgrounds (educational researchers, nurse, and physicians) enabling both internal and external perspectives of the phenomenon under study. Secondly, by collecting data using both questionnaire and interviews and by discussing the emergent findings repeatedly in the research team, transparency and credibility of the analysis process was ensured.^32^

### Findings

Our findings comprise five themes, each capturing a type of narrative that caused students discomfort and emotional reactions. The majority of the narratives concerned situations in the clinical environment. The narratives concerned situations regarding a) confronting illness and death, b) witnessing unprofessional behaviour, c) dilemmas regarding different views on the treatment of patients, d) relating to patients as individuals and not diagnoses, and e) using patients for the students’ own learning. Each theme is described in more detail below and illustrated with quotes.

### Confronting illness and death

A majority of the narratives concerned students confronting illness and death. It was a strong, often overwhelming, experience to meet patients with severe diseases, who were suffering or were in pain, and sometimes dying.

The experience of confronting death and the dead body was emotionally strong and evoked existential reflections on life and death for some students. The narratives from medical students included situations from anatomy dissection and/or autopsy as well as the death of a patient, while the nursing students only mentioned narratives from the clinical environment. Both medical and nursing students felt unprepared to deal with these situations at first. During their education, students met patients with severe diseases who were suffering. These experiences affected students, and they felt unease, sorrow, and empathy. Other cases concerned dramatic events where the patient’s condition suddenly deteriorated. These narratives described a more stressful, chaotic situation. Patients sometimes reacted emotionally to a treatment or to information about their disease. The students, and in one case the supervisor, found it difficult to keep calm and not become affected by the patient’s reaction. Medical and nursing students’ narratives were very similar concerning confronting illness and death. The quote below represents narratives concerning a traumatic event.

“A man was choking on a piece of cinnamon bun. His airways were blocked. In connection with this, he also had a heart attack. It was horrible to see the panic in his eyes and how his face turned more and more blue. That is an image I will never forget. I stood as if frozen long after the ambulance had taken him away. I thought he was just choking to death right in front of me.” (25, nursing student, final year)

This narrative described the horror the student felt when witnessing a patient choking. The situation was dramatic, and the student found herself in a state of paralysis, even after the patient was taken to the emergency room. The experience was quite overwhelming for the student.

“The experiences in Africa were very helpful for me. At first, you are completely overwhelmed by everything… […] dismayed by a lot of what you see.” (Medical student, final year, interview)

This medical student described her experiences from an exchange visit to a public hospital in Africa, where she saw many severely ill patients that affected her deeply.

### Witnessing unprofessional behaviour

This theme concerned unprofessional behaviour by supervisors or other healthcare professionals. The behaviour was often directed towards patients or the supervisor (a young intern). In some narratives, the behaviour was directed towards the student. Both nursing and medical students’ narratives revealed that they witnessed unprofessional behaviour during their clinical placements. The students expressed feelings of unease and sometimes anxiety or guilt. They felt that the behaviour was unethical, unworthy and problematic. One type of behaviour that students found unprofessional was a lack of empathy. The essence of these narratives was that healthcare professionals, sometimes the supervisor, did not meet the patient’s needs, for instance by neglecting to do certain things for the patient, which the student thought was humiliating for the patient. Other narratives concerned a nonchalant attitude on the part of healthcare professionals, who behaved badly towards patients, other healthcare professionals or students. The students’ narratives revealed a difference between nursing and medical students; some nursing students acted and tried to compensate for the unprofessional behaviour that they witnessed; medical students did not. The following quotes are examples of such narratives.

“A patient wanted to pee in the bedpan, and the lifting of the patient was (in my opinion) brutal and rough. Afterwards, the patient was not lying comfortably in the bed, and I went out and asked the staff how to make it more comfortable for the patient. I got the answer: ‘Tell him that you will come back later, and then leave!’ I was working with a nurse’s assistant, and the nurse was not present at the time, so I chose to go in again and together with the patient’s relatives we made it comfortable for the patient, helping him to sit up in bed.” (24, nursing student, final year)

“There are many typical situations that are difficult in various ways, for instance during consultations when the supervisor does not treat the patient correctly, but you as a ‘colleague’ are expected to back up or at least not to contradict and thereby undermine the doctor’s message to the patient.” (37, medical student, final year)

The first quote represents a situation where the student compensated for the lack of empathy and unprofessional behaviour towards the patient. The student was concerned for the patient’s well-being and, together with relatives, she helped the patient, not complying with the advice from healthcare professionals. The second quote illustrates the situation where a medical student is feeling the pressure to back up a supervisor, even though this contradicts his own values.

### Experiencing dilemmas regarding patient treatment

In this theme, the students experienced a dilemma regarding the care or treatment of patients. In some narratives, the student’s opinion contradicts their supervisor’s; in other narratives, their supervisor and another more experienced healthcare professional had different opinions. These situations were challenging for students, left them confused about the priorities that chosen and why certain treatments were administered. Students experienced feelings of unease, confusion, frustration, and in some cases guilt. Dilemmas that students experienced could concern the ethical aspects regarding patient treatment. Sometimes, students could not see the benefits of a certain treatment for a patient and therefore questioned the decision for ethical reasons. In other cases, students had different opinions regarding patient treatment. Students wanted to do what was best for the patient, and, when other healthcare professionals treated the patient or acted in a way that students disagreed with, they felt frustrated. A few narratives concerned coercive care. Coercive psychiatric care, where patients were given treatment against their will and without consent, evoked strong emotional reactions in students and reflections on ethical issues. The quotes below are narratives where students experienced dilemmas regarding patient treatment.

“A 90-year old woman came into the operating room with a ruptured aortic aneurysm. The surgeon and I were the first to arrive, and I got to assist. It was a very bloody surgery, and around 10 bags of blood were used. […] Later during the procedure, she (the patient) suffered a cardiac arrest, and the surgeons needed to conduct a cardiopulmonary resuscitation and managed to start the heart. They covered the operation area […] and could not finish the surgery that day. I did not get any follow-up, and no one asked me how I felt. I don’t know what happened to her, but it all felt macabre, and the surgery was probably unnecessary since she hadn’t a very good chance of surviving. It was a rough atmosphere. […] It would have been good to discuss the ethical aspects of performing this surgery on a 90-year old person who was severely ill.” (43, medical student, final year)

“I (as a student) wanted to take the blood sample (prioritise that patient) […]. But my supervisor said no, which resulted in several hours of waiting for the patient [...]. The patient became impatient and was very hungry! Everything felt like it was my fault and I felt guilty.” (8, nursing student, final year)

These quotes illustrate the feelings of guilt and unease when students experience dilemmas regarding patient treatment.  Another example is a medical student whose supervisor prescribed medication causing addiction to a patient with known substance abuse. “I think it is very wrong to prescribe that kind of drug to a patient with a known addiction problem” (medical student, final year, interview). The students do not always know why certain medical decisions are made, and often they do not get the opportunity to discuss this with their supervisor afterwards.

### Relating to patients as individuals and not diagnoses

The main issue in this theme is situations when students related to the patient as a human being and become too involved emotionally. In doing so, they also thought about the patient having a family, friends, job, etc., and they found it more difficult to maintain a professional attitude to the patient. Students became more emotionally affected, often overwhelmed, and they felt close to the patient and their family. Sometimes the patient’s story reminded them of their own personal experiences, and handling both the patient’s and their own emotions became difficult. In other narratives, the patient’s life story affected them deeply. The patient’s vulnerability became clear to them. It seems that the students confused their own emotional reactions with the emotions of the patient. In our findings, both nursing and medical students related to their own personal experiences, while only medical students’ narratives described being deeply touched by a patient’s life story.

“It was during my clinical practice in the second year when there was a situation with a dying patient. Just two months before, my grandmother had died, which was hard for me. This patient, and their relatives reminded me a lot of my own family and the time when grandmother was so ill. I thought it was very difficult to ignore my own feelings, even if that was considered ‘unprofessional’. I could not handle the situation in a good way, so I told my supervisor that I did not want to have so much contact with that particular patient.”  (5, nursing student, final year)

“During the psychiatric clinical placement, there was a man who, through an interpreter, told us in quite some detail about how he lost his wife and children in a horrible way during a war situation and the nightmares he now experienced. Both the interpreter and I cried. It was a very strong emotional experience, and I still get tears in my eyes just thinking about it.” (62, medical student, final year)

These two quotes illustrate narratives where students had difficulties in managing their own emotions in patient encounters, and this led to difficulties upholding a professional attitude in the situation. Another example of this is a medical student who met a patient whom the healthcare professionals involved suspected of being a victim of trafficking.

“And you know that you are sending her home to… something that is probably awful. And it was very difficult to deal with.” (medical student, final year, interview)

### Using patients for own learning

This theme concerns students experiencing a dilemma when they felt that they were “using” patients for their own learning. This dilemma is found in narratives from both medical and nursing students. Sometimes, when dissecting or examining a patient, students felt it was unworthy and wrong to explore vulnerable patients just for the benefit of their learning. This theme was not as strong as the other themes, but there were a few narratives that concerned this dilemma. The following quote is an example of such situations.

“[It was] A patient in the haematology department who did not have a promising prognosis. He was not ‘my’ patient, but I was pushed into his room because he had an interesting medical status […]. It felt very awkward and false to go in to see this patient, who was staring blankly at the wall and was clearly depressed, just to broaden my medical experience.” (36, medical student, final year)

The narrative above illustrates the feeling of unease when the student was pushed to meet a patient, primarily to learn rather than to participate in the care of the patient. The student clearly found himself in a dilemma.

### Dealing with emotionally challenging situations

Two overarching themes describe the way students in our study dealt, or wanted to deal, with emotionally challenging situations during their undergraduate education: the need for support from others and getting used to the situation.

In our study, when they needed to talk about their experiences, the majority of both medical and nursing students talked to their peers. Some students had supervisors that they could turn to for support; others were left alone to handle their experiences. The overall picture that our findings point to is that it is important for students to have people that they can confide in when they experience something out of the ordinary. Trust and confidence were described as important when the students talked about their emotional experiences. Also, it seems that the support should be timely, i.e., the same day or the days after. Talking to others made students realise that they were not alone in feeling this way, which was a confirmation and a relief. It seems that students needed to understand what had happened during the entire situation, i.e. medically and why certain treatments were chosen. That made it easier to deal with the experience of confronting illness and death as well as dilemmas regarding patient treatment. The following quote is an example of a narrative where a student talked to and received support from a supervisor.

“I was in the maternity clinic [...] and had a resident physician as a supervisor. We received information that a woman was seeking urgent care for decreasing foetal movements. We went in to see her and her husband and perform an ultrasound. The baby had died in the uterus. It became very chaotic in the room when the woman reacted very strongly. It was particularly difficult since the woman only knew a little Swedish and there was no interpreter there. So, her sad and shocked husband had to act as an interpreter. I was deeply affected and sad and wished that I could go away. […] I talked briefly to my supervisor afterward, mainly from a medical point of view, but we also talked about how it was a difficult situation and what it is like to break such bad news. […] I often think about that woman, and how unfair it all felt.” (57, medical student, final year)

In situations where the clinical supervisor or other healthcare professionals that students meet during clinical practice acted unprofessionally, it seemed more common to talk to peers or family members than to other healthcare professionals on the clinical placement. However, some students did not talk to anyone about what they experienced. The quote below describes a situation where a student experienced unprofessional behaviour from healthcare professionals and how that affected her.

 “I was on the oncology ward where I felt that the patients’ feelings and mood were being ignored and (they) only dealt with the physical (aspects). Everyone (on the staff) was too busy to see the patients. For me, as an outsider, the whole atmosphere was quite exhausting and affected my state of mind. […] I dealt with the whole situation by talking to my partner and friends. The difficult part was not the patients, but the reluctance among the healthcare professionals to meet the patients’ emotional needs – they didn’t have time or energy to be empathic.” (3, nursing student, final year)

In addition to talking to other people, students got used to the situations that caused emotional unpleasantness and in time felt more secure in their own ability to deal with these kinds of situations. The students mentioned that “it is much easier now than in the beginning”. Also, feeling more secure seemed to be related to students learning to trust that they had done all they could in these situations.

“I think that, when you have seen a few [palliative] patients, and you have talked about that, then it is like: ’OK, now I know what to do’, and I feel more confident because it is similar with all palliative care patients. Caregiving is the most important. […] So, it is more that you find your confidence in the situation.” (Nursing student, final year, interview)

“One strategy has been to […] not to protect myself from it (difficult emotions), and not just to think: ‘No, this is too difficult and unpleasant, I’ll turn away from that’, but actually do the opposite. […] I think it is about seeing (these situations) several times, talking to patients about how they are feeling. […] I don’t know if it is a habit or if you get detached or if you get used to it, but you… just become more professional.” (Medical student, final year, interview)

The quotes above illustrate that the process of getting used to emotionally challenging situations seemed to happen without students knowing precisely how, but students learned what to expect, and this seemed to help them manage the situation and their own emotions.

## Discussion

Our findings suggest that there is a variety of situations that students find emotionally challenging, in pre-clinical and, for the most part, in clinical environments. Some of the narratives concerned situations that are part of the formal curriculum, i.e., confronting illness and death, dilemmas regarding treatment, and close contact with various aspects of human life and patients’ life histories. Other narratives focused on issues that concerned the informal or hidden curriculum, such as various unprofessional behaviours by healthcare professionals. Moreover, medical and nursing students’ experiences were similar, despite their different educational programmes (with different focus, knowledge, and aims) and the circumstances of their clinical placements.

A large part of the narratives in our study concerned students confronting illness and death. Seeing a dead patient, or witnessing suffering and pain were strong experiences that affected students emotionally. Students felt unease and sorrow and were unprepared to deal with these situations at first. This is in agreement with previous studies.^2^^,^^7^^-^^10^^,^^23^^,^^33^ Caring for, and sometimes curing, patients and witnessing suffering and pain are inevitable parts of medical and healthcare practice. Students need to learn how to manage their own reactions in situations that are emotionally challenging. Students’ narratives also focused on dilemmas regarding patient treatment. In some cases, they did not understand the priorities chosen; in other cases, they felt that they would have acted differently. Students felt frustration, unease, and confusion. These situations evoked reflections on ethical aspects of healthcare and medicine and the fact that professionals often have to deal with uncertainties.

A few narratives in our study concerned the students’ dilemma about using patients for their own learning. Students experienced feelings of guilt when using patients for their own learning, and they could relate to the patients’ vulnerability in the situation. Others have also described this finding.^14^^,^^33^ In our study, only a few of the narratives concerned dilemmas about using patients for own learning. However, other researchers found this to be a common experience.^14^ Learning from patients is part of medical and healthcare professional learning, and it is important to discuss with students how to manage the guilt that this may trigger, and how to ensure valid patient consent. If the students know that the patients have agreed to let the student examine them or take an active part in patient care, it may be less of a dilemma for the student.

Studies have found that, in the situations described above, a survival strategy for some students, is to distance themselves emotionally and to suppress their emotions and become more detached.^2^^,^^23^^,^^34^^,^^35^ Another aspect of the struggle to balance closeness and distance in relation to patient care is situations where students become overwhelmed by emotions. In our findings, the situations students mentioned were when a patient’s situation reminded them of their own personal experiences or when a patient’s life story affected them so deeply that the balance between closeness and distance was difficult to maintain. In these circumstances, the professional attitude was hard to uphold, and students seemed to find it difficult to distinguish their own emotions from empathy for the patient. Students will most likely experience similar situations in the future. It is, therefore, important for them to learn to be aware of their own as well as their patients’ emotions. Managing and responding to emotions is part of good patient care.^1^

Caring for people with severe illnesses and dealing with uncertainty are central aspects of medical and nursing education and the students’ future profession. The situations discussed above are aspects that are part of the formal curriculum. In order to help students reflect on their experiences from the clinical placements, both the nursing and medical curriculum in our study provided opportunities for students to discuss and reflect on aspects of clinical work and their professional development in organised seminars or reflection groups. Our findings point to the need for additional support. Students’ stories revealed that they dealt with their experiences by talking primarily to their peers, and in some cases to their supervisor. It also seems that these conversations were informal and close in time to the actual event or situation, suggesting the need for more immediate support complementing the reflection seminars/groups mentioned above. Moreover, in several narratives, where students were clearly affected emotionally, they were left alone to deal with their experiences. Thus, our findings together with those of previous literature, suggest that, when they find themselves in situations that affect them emotionally, students need support and opportunities to talk to someone they trust and to reflect on their experiences.^36^ However, support in the form of reflection seminars with senior physicians offered as a part of the formal curriculum, or support in the form of informal conversations with peers and supervisors are different and probably serve different purposes regarding the students’ learning and well-being. We believe both types of support are needed.

In addition to managing by talking about their experiences, students seemed to get used to these situations that caused them strong negative emotions. Their narratives showed that they felt more secure and confident, knew what to expect, and became more able to handle the situation. This process of getting used to emotionally challenging situations may occur due to either a desensitisation^5^ or perhaps a shift in focus, from the overwhelming emotions to the practical clinical work, or learning to balance closeness and distance.^2^ Learning to manage emotionally challenging situations is part of the professional development that students undergo during their training. This study points to the need for more research in order to better understand how students get used to and learn to manage their emotions in medical training.

The students’ narratives in our study also concerned situations where healthcare professionals behaved unprofessionally, i.e., demonstrated lack of empathy or a nonchalant attitude towards patients or students – findings confirming previous studies.^11^^,^^13^^,^^14^^,^^37^ Thus, observing the unprofessional behaviour of healthcare professionals is not an uncommon experience for medical and nursing students during their clinical placements. Also, Doja et al.^38^ found not only the “existence of unprofessional behaviours but also the fact that these behaviours were tolerated on a regular basis”. These behaviours from healthcare professionals are part of the clinical environment and, from the students’ perspective, become part of the hidden curriculum and subsequently influence their learning.^18^^,^^21^^,^^24^^,^^38^ It is possible that these situations are more problematic than situations concerning patients’ diseases and treatments, discussed above. For instance, who do students talk to when their supervisor or, in the future, their colleague behaves unprofessionally? Students are dependent on their supervisors, and it may, therefore, be especially difficult to talk to them about these issues. Furthermore, what influence does witnessing unprofessional behaviour by healthcare professionals have on students’ professional development? This may foster a sense of powerlessness in students, and a tension between idealised views of healthcare practice and what they experience during clinical placements.^35^^,^^38^ Students meet and are required to engage in two different communities of practice:^39^ one represented by the formal curriculum with certain ideas about professional values, norms and practice, and the other represented by the informal curriculum in the clinical environment with sometimes contradictory values, norms and practice. It seems difficult for students to bridge these different communities, and studies suggest that the informal curriculum may have more influence.^18^^,^^21^^,^^22^

### Limitations

There are some inherent limitations in this study. This study explored students’ experiences of emotional challenges, and data was gathered mainly using open-ended questionnaires. The advantage of using open-ended questionnaires is that it is an efficient way to gather data from many informants when the time is limited. However, the disadvantage is that the data is not as rich and nuanced as when using interviews and asking follow-up questions is also more complicated. To compensate for the limitations of the questionnaire, individual interviews were conducted. The questionnaire data together with the interviews gave us a broad understanding of students’ experiences. The interview data fitted well with the findings from the questionnaires. We used self-reported data in this study; however, investigating students’ own experiences and how they felt is only possible by asking them about it. Another limitation of the study was that it was conducted in a specific context, at one university. The usefulness of our findings to other contexts depends on the similarities of the contexts. However, our findings are in agreement with and complement those from previous research.

### Implications for practice

This study shows that both medical and nursing students experience a range of situations during their undergraduate education which they find emotionally challenging, in pre-clinical and particularly in clinical environments. The students often feel unprepared to manage these situations, and they need support and opportunities to talk to trusted peers and supervisors. It seems important for students to reflect on what they have experienced and to discuss how to manage their emotions. Our findings also point to the need for awareness, of both medical educators and clinical supervisors, that students may experience emotionally difficult situations during their undergraduate education, and that they need various kinds of support. This support could take the form of organised seminars, spontaneous conversations and an attitude that emotions are not only allowed but normal, in the medical practice. Also, students should be trained and encouraged to talk to each other about their experiences, which may, in the long run, contribute to a change in the informal curriculum influencing the clinical environment.

## Conclusions

Despite the quite different knowledge, experiences and conditions for medical and nursing students, where medical students have short clinical placements, our findings suggest that their experiences were very similar. Students’ narratives concerned situations related to patients’ illness and death, witnessing unprofessional behaviour, dilemmas regarding patient treatment, relating to patients as human beings, and using patients for their own learning. The students’ narratives revealed that they often felt unprepared to manage emotionally challenging situations. Support and opportunities to talk to trusted peers and supervisors were important. Our findings suggest that students got used to these situations and felt more confident with time and experience, but more research is needed to understand how students learn to manage these kinds of situation and how these experiences affect their empathy, their mental health, and their own ideas and development of professionalism.

### Acknowledgments

We wish to express our gratitude to the students who participated in this study. The study was supported financially through Swedish Research Council (Grant number 2013-2310). We would like to thank Dr Linda Barman for valuable comments on an earlier version of this paper.

### Conflict of Interest

The authors declare that they have no conflict of interest.
